# Two‐Step Design Rule for Simultaneously High Conductivity and Seebeck Coefficient in Conjugated Polymer‐Based Thermoelectrics

**DOI:** 10.1002/advs.202409382

**Published:** 2024-11-08

**Authors:** Zelong Li, Wei Fu, Dorothea Scheunemann, Xiaoran Wei, Maximilian Litterst, Priya Mariam Viji, Yong Cui, Jianhui Hou, Junhui Tang, Ziqi Liang, Zehua Qu, Martijn Kemerink, Ruiqian Guo, Guangzheng Zuo

**Affiliations:** ^1^ Institute for Electric Light Sources School of Information Science and Technology Fudan University Shanghai 200433 P. R. China; ^2^ Institute for Molecular Systems Engineering and Advanced Materials Heidelberg University Im Neuenheimer Feld 225 69120 Heidelberg Germany; ^3^ State Key Laboratory of Polymer Physics and Chemistry Beijing National Laboratory for Molecular Sciences CAS Research/Education Center for Excellence in Molecular Sciences Institute of Chemistry Chinese Academy of Sciences Beijing 100190 P. R. China; ^4^ Department of Materials Science Fudan University Shanghai 200433 P. R. China; ^5^ State Key Laboratory of Molecular Engineering of Polymers Department of Macromolecular Science Fudan University Shanghai 200433 P. R. China

**Keywords:** conductivity, energetic disorder, fluorine substitution, organic thermoelectrics, seebeck coefficient

## Abstract

The trade‐off between enhancing conductivity (σ) through doping while concurrently observing a reduction in the Seebeck coefficient (*S*) presents a key challenge in organic thermoelectrics. Here, a two‐step structural design strategy is developed, where the first step enhances the backbone planarity which enhances the conductivity by an improved ordering of conjugated polymers (CPs). The second step, which is fluorination of the backbone, improves the Seebeck coefficient by the controlled induction of energetic disorder, stemming from the fluorine's disruption of the homogeneous electrostatic potential across the CP backbone. This strategy is applied to two series of donor‐acceptor (D‐A) types of CPs based on the BDT donor unit and BDD and TT as acceptor units, respectively. A maximum power factor (*PF*) over 155 (142.7 ± 12.7) µW m^−1^ K^−2^, coupled with *S* ≈202 (194.6 ± 7.6) µV K^−1^ is achieved, leading to up to 32‐fold enhancement of the *PF* compared to the initial non‐planar and non‐fluorinated polymer. This study provides valuable conceptual insights for designing CPs with high conductivity and Seebeck coefficient.

## Graphical abstract

A two‐step structural design strategy is proposed and applied to conjugated polymers for achieving both high conductivity and a high Seebeck coefficient. The first step enhances the backbone planarity, enhancing the conductivity by improved ordering of conjugated polymers. The second step fluorinates the backbone, improving the Seebeck coefficient by the controlled induction of energetic disorder.

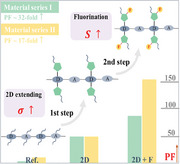

## 1 Introduction

Conjugated polymer (CP) ‐based thermoelectric (TE) materials have received considerable attention in recent years, mainly due to their low thermal conductivity and potential large‐scale processability compared to conventional bulk thermoelectric materials.^[^ ^1^, ^2^, ^3^, ^4^, ^5^, ^6^, ^7^, ^8^, ^9^ ^]^ Typically, the performance of TE materials is evaluated based on the figure of merit (*ZT*) value, $ZT=\frac{\sigma S^{2}}{\kappa }T$, where σ is the electrical conductivity, *S* is the Seebeck coefficient, κ is the thermal conductivity and *T* is the temperature. Owing to the intrinsic low thermal conductivity of conjugated polymers, an efficient TE material is primarily determined by σ and *S*. However, the electrical conductivity of intrinsic conjugated polymers is generally low, originating from their disordered nature and ultra‐low charge concentration. A way to circumvent this issue and enhance electrical conductivity is molecular doping.^[^ ^10^, ^11^, ^12^, ^13^ ^]^ For instance, Zhong et al.^[^ ^14^ ^]^ investigated the influence of the p‐type dopant on the TE properties of a prototypical CP, P3HT, including F_4_TCNQ, FeCl_3,_ and magic blue (MB), yielding a range of conductivities 200–3000 S cm^−1^, accompanied by Seebeck coefficients of 10–50 µV K^−1^. Focusing on phase‐selective incorporation of certain dopants, Dash et al.^[^ ^15^ ^]^ demonstrated that spontaneous modulation doping can occur in polymer semiconductors at low dopant concentration, and achieved an ultra‐high σ  ≈ 9700 S cm^−1^, coupled with *S* ≈15 µV K^−1^ for PBTTT‐C_12_ at low MB (but not F_6_TCNQ) concentration of 0.6 mm.

While the above strategies prove successful in increasing conductivity, they usually lead to a decrease in the Seebeck coefficient at the same time. Glaudell et al.^[^ ^10^ ^]^ found an empirical −1/4 power‐law relationship between the Seebeck coefficient and the conductivity of p‐type Organic Semiconductors (OSCs), which limits the *PF* to *PF*  =  σ*S* ^2^∝σ^1/2^. Later, Abdalla et al.^[^ ^16^ ^]^ demonstrated that the empirical −1/4 power law can be attributed to the additional density of states (DOS) induced by Coulomb traps stemming from dopant counterions at high doping concentrations. More specifically, charge and energy transport for a disordered system is governed by the Fermi energy (*E_F_*), that is determined by the charge carrier concentration and the transport energy (*E_tr_*) that is determined by the shape and magnitude of the DOS.^[^ ^16^, ^17^ ^]^ Then, *S*∝(*E_tr_* − *E_F_*) holds for the Seebeck coefficient, while for the conductivity σ∝exp (*E_F_* − *E_tr_*). Hence, both σ and *S* are determined by the energy difference Δ*E*  = *E_tr_*  − *E_F_* and a smaller Δ*E* corresponds to a larger σ but a smaller *S*.

Building upon the above, it is challenging to reconcile the trade‐off between conductivity and the Seebeck coefficient solely through changes in dopants and doping methods. Electrical conductivity (σ  =  *n* · *e* · μ) is decided by the charge density (*n*) and mobility (μ), where *e* is the elementary charge. Doping primarily increases *n* but this invariably leads to the introduction of Coulomb traps, negatively affecting the DOS by broadening it. To attain high conductivity while minimizing the need for high dopant concentrations and mitigating the decrease in the Seebeck coefficient, one potential avenue is to rationally design CPs with high mobility, which increases σ without increasing *n*, and simultaneously controlling the degree of energetic disorder to maximize the product σ*S* ^2^. It should be noted that carrier mobility and charge transport behavior in doped systems may deviate from intrinsic material due to state filling, doping, charge screening, and so on.^[^ ^16^, ^18^, ^19^, ^20^ ^]^

Among the molecular design strategies for CPs with high mobility, one is the construction of highly planar, quasi‐2D conjugated polymers, which is beneficial for the long‐range charge transport and carrier mobility, due to increased conjugation along the polymer backbone and increased interchain π–π overlap. This concept was first introduced and applied by Hou et al.,^[^ ^21^ ^]^ who utilized 2D conjugated polythiophenes (PTs) in organic solar cells (OPV). Subsequently, they introduced this 2D design strategy to additional CPs, and their findings demonstrated a significant enhancement in the hole mobilities of CPs, leading to a notable improvement in the performance of OPV.^[^ ^22^ ^]^ For instance, Hou et al. introduced alkylthienyl substituents onto benzo[1,2‐b:4,5‐b'] bithiophene (BDT) units to create the 2D conjugated polymer PBDTTT‐C‐T, achieving a high hole mobility of 0.27 cm^2^ V⁻¹ s⁻¹, which is three orders of magnitude higher than the 1D polymer PBDTTT‐C (5 × 10⁻⁴ cm^2^ V⁻¹ s⁻¹).^[^ ^23^ ^]^ Similarly, other studies also have indicated that transitioning to 2D conjugated structures improves hole mobilities.^[^ ^24^, ^25^, ^26^ ^]^ Figure S22 and Table S4 (Supporting Information) summarize these findings, as presented in Supporting Information. In more recent work, Zhou et al.^[^ ^27^ ^]^ adopted this 2D strategy and synthesized two CPs of P(BDT‐EDOT) and P(BDTTT‐EDOT) by using alkylthienothiophene groups instead of alkoxy groups on the BDT unit. They immersed the films in ferric chloride (FeCl_3_) solution (20 mg mL^−1^) and achieved σ ≈0.81 and 1.64 S cm^−1^ and the corresponding PF ≈0.37 and 2.19 µW m^−1^ K^−2^ for P(BDT‐EDOT) and P(BDTTT‐EDOT) at room temperature, respectively. They attributed the improved performance of P(BDTTT‐EDOT) to its larger conjugated plane and better interchain π–π overlap compared to P(BDT‐EDOT), which enhances charge transport and increases carrier mobility. Later in the same group, Li et al.^[^ ^28^ ^]^ modified the acceptor unit of EDOT to DPP to construct the materials P(BDT‐DPP) and P(BDTTT‐DPP). They obtained an optimal PF ≈6.5 µW m^−1^ K^−2^ for P(BDTTT‐DPP), which was 26 times higher than that of P(BDT‐DPP). Previously, Li et al. have synthesized and investigated the P(BDT‐DPP) and P(BDTTT‐DPP), finding a high hole mobility of 2.4 × 10⁻⁴ cm^2^ V⁻¹ s⁻¹, which is ≈1.1 × 10⁻⁴ cm^2^ V⁻¹ s⁻¹ for PBDT‐DPP.^[^ ^29^ ^]^

Several studies have indicated that the inclusion of fluorine atoms in CPs can result in a potentially more planar backbone structure through inter‐ and intrachain interactions,^[^ ^30^, ^31^, ^32^, ^33^, ^34^, ^35^, ^36^ ^]^ resulting in an enhancement in mobility. This strategy has found widespread use in organic field‐effect transistors (OFET) and OPV. For example, Price et al.^[^ ^34^ ^]^ synthesized fluorinated polymer PBnDT‐FTAZ and found its hole mobility ≈6.76 × 10^−5^ cm^2^ V^−1^ s^−1^, which is 20 times higher than that of the corresponding non‐ fluorinated PBnDT‐HTAZ.

Here, we demonstrate that it is possible to unite these two strategies into a universal two‐step molecular design strategy aimed at simultaneously achieving high conductivity and Seebeck coefficient. First, we extend the 2D conjugated structure, followed by introducing fluorine (F) substituent on the backbone. Utilizing benzo[1,2‐b:4,5‐b'] bithiophene (BDT) as the base unit, we construct CPs such as PBDB‐O, PBDB‐T (2D), and PM6 (F‐substituent). We investigate the conductivity and Seebeck coefficient of polymers using sequential doping with FeCl_3_. PBDB‐T shows enhanced conductivity exceeding 65.2 ± 2.8 S cm^−1^ due to improved molecular packing and increased dopant intercalation from the 2D conjugated structure. The additional introduction of F‐substituents in PM6 increases the Seebeck coefficient from 87.0 ± 1.0 to 123.5 ± 0.5 µV K^−1^ due to induced disorder disrupting charge distribution while only moderately reducing σ. With our two‐step strategy, PM6 achieves a remarkable 32‐fold enhancement in power factor (PF) exceeding 85.2 ± 3.4 µW m^−1^ K^−2^. Similar trends are observed in the PTB7‐Th series, reaching a maximum PF exceeding 155 (142.7 ± 12.7) µW m^−1^ K^−2^ with S ≈202 (194.6 ± 7.6) µV K^−1^. Our work provides insights into CP design rules for enhancing conductivity and Seebeck coefficient simultaneously, offering a practical strategy for improving CP thermoelectric performance through structural design.

## 2 Results and Discussion

**Figure 1:**
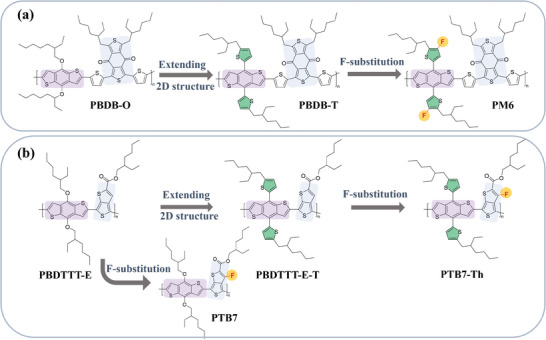
Illustration of the evolution of chemical structures via two‐step design rule for a). PM6 series with polymers PBDB‐O, PBDB‐T, and PM6, and b). PTB7‐Th series with polymers PBDTTT‐E, PTB7, PBDTTT‐E‐T and PTB7‐Th.

The materials investigated in this study are depicted in **Figure** **1**, illustrating the evolution of their chemical structures based on the two‐step design rule. Benzo[1,2‐b:4,5‐b'] dithiophene (BDT) serves as the base donor (D) unit for constructing two distinct series of donor‐acceptor (D‐A) type CP materials. One series involves the selection of 1,3‐bis(thiophen‐2‐yl)‐5,7‐bis(2‐ethylhexyl) benzo[1,2‐c:4,5‐c'] dithiophene‐4,8‐dione (BDD) as the acceptor (A) unit, leading to the referenced material PBDB‐O. Exchanging the alkoxy groups of the BDT unit to alkyl‐thiophene groups results in the 2D extended structure of PBDB‐T. Finally, PM6 is obtained by the introduction of fluorine atoms in the thiophene ring of the BDT side chains. The other series features the choice of (2‐ethylhexyl) carbonyl] thieno[3,4‐b] thiophene (TT) as the A unit, resulting in the PTB7‐Th series CPs comprising PBDTTT‐E (as reference), PBDTTT‐E‐T (2D extended), and PTB7‐Th (2D extended and fluorination). As shown in Figure 1b, we also used material PTB7 with fluorination while without 2D extending, specifically examining the impact of specific steps of the proposed two‐step design rule. To address the limitations associated with conventional bulk doping on morphology, we adopted a sequential doping method. This involved spin‐coating FeCl_3_ solution onto pre‐prepared pristine CP films across a wide range of FeCl_3_ concentrations. Further experimental details are provided in the Experimental Section.

**Figure 2:**
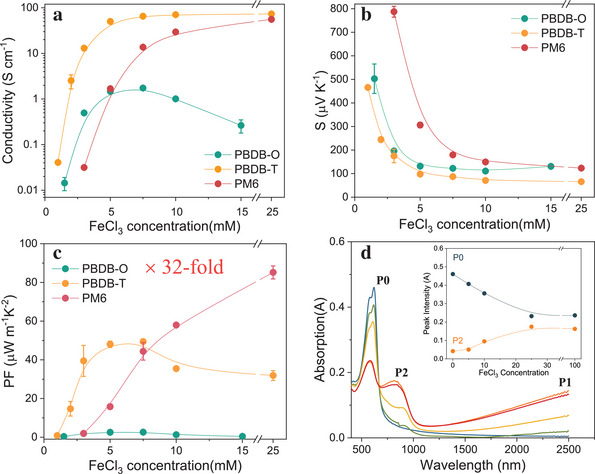
Thermoelectric characteristics of doped CPs as a function of FeCl_3_ concentration for a) conductivity (σ), b) Seebeck coefficient (*S*), and c) power factor (*PF*). d) the UV–vis–NIR absorption spectra of pristine and doped PM6, the inset is the curve of P0 and P2 absorption intensity as a function of dopant concentration.

The thermoelectric properties were evaluated by measuring the conductivity and Seebeck coefficient on lateral (in‐plane) devices, following the methods described in detail in previous work.^[^ ^17^, ^37^ ^]^ **Figure** **2** presents the results alongside the corresponding power factor (*PF*  =  σ*S* ^2^). For PBDB‐O doped with 7.5 mM FeCl_3_ solution, we observed a conductivity σ ≈ 1.74 ± 0.04 S cm^−1^ and a corresponding *S* ≈ 122.3 ± 2.8 µV K^−1^, resulting in a *PF* of ≈2.60 ± 0.18 µW m^−1^ K^−2^. Upon composing PBDB‐T with a more 2D conjugated structure using the thiophene ring, the conductivity surged to over 65.2 ± 2.8 S cm^−1^, while the corresponding Seebeck coefficient decreased from 122.3 ± 2.8 to 87 ± 1 µV K^−1^. Further modification of the CPs structure via F‐substitution on the thiophene ring led to a recovery of the Seebeck coefficient of up to 123.5 ± 0.5 µV K^−1^(PM6) while the conductivity dropped only mildly, especially at higher doping levels. We achieved an optimal *PF* exceeding 85.2 ± 3.4 µW m^−1^ K^−2^ for PM6, representing a 32‐fold improvement compared to the reference material PBDB‐O.

It is noteworthy that the conductivity and Seebeck coefficient for both PBDB‐T and PM6 remained relatively constant beyond 10 mm FeCl_3_ concentration, as depicted in Figure 2a,b, respectively (Detailed results for PM6 doped with FeCl_3_ over a wider doping range are provided in Figure S7, Supporting Information). This observation suggests that the sequential doping method reaches its doping efficiency limitation already at intermediate FeCl_3_ concentrations. To confirm this assumption, we conducted absorption spectra measurements of PM6 across FeCl_3_ concentrations ranging from 0 to 100 mm, as shown in Figure 2d. The results indicate only minimal changes in absorption intensities for both the pristine peak (≈600 nm, P0) and the doping‐induced peak (≈800 nm, P2) at FeCl_3_ concentrations exceeding 10 mm. This trend suggests that the sequential doping ability saturates at high concentrations for such 2D‐constructed CPs, supporting our hypothesis regarding doping limitation. We speculate that this is because the dopants may encounter difficulty penetrating deeply into the films due to the increased ordering of CPs by extending the 2D structure, particularly when employing the sequential doping process. More absorption spectra for the other CPs are shown in the Section S1 (Supporting Information).

**Figure 3:**
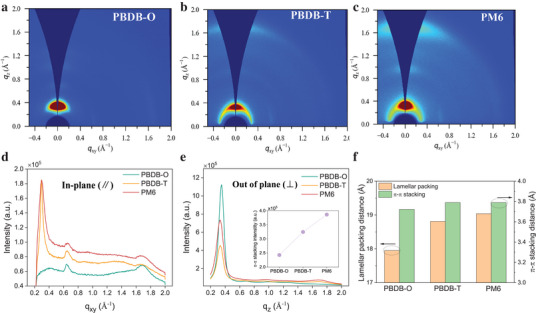
2D‐GIWAXS profiles of a). PBDB‐O, b). PBDB‐T and c). PM6 and the corresponding d). in‐plane (∥), e). out‐of‐plane (⊥) scattering profiles. f) lamellar packing distance (100) and π–π stacking distance (010) for pristine PBDB‐O, PBDB‐T, and PM6, respectively.

To rationalize the influence of molecular structure on charge transport behavior via the two‐step design rule, we inspected the film morphology using the grazing incidence wide‐angle X‐ray scattering (GIWAXS) technique to investigate the molecular packing motifs of the three pristine CPs. The 2D‐GIWAXS images and the corresponding in‐plane (∥) and out‐of‐plane (⊥) scattering profiles are shown in **Figure** **3**a–e, respectively. It is unveiled that both pristine PBDB‐T and PM6 exhibit a high degree of ordering along the *q_xy_* direction with face‐on orientations, indicating a more planar structure and improved molecular packing in polymers with a 2D backbone. As shown in Figure 3f, for PBDB‐O, the lamellar packing (100) distance is calculated from the peak to be 17.95 Å (*q_xy_* =  0.35 Å^−1^), along with a π–π stacking (010) distance of 3.72 Å (*q_z_* =  1.69 Å^−1^). After extending the 2D conjugated structure, PBDB‐T (2D) shows an increased lamellar packing distance of 18.81 Å (*q_xy_* =  0.334 Å^−1^), and a slight increase in π–π stacking distance from 3.72 to 3.79 Å (*q_z_* =  1.66 Å^−1^). This increase may be attributed to the larger thiophene ring and branched side chains on the BDT unit, which lengthens the side chains and induces a dihedral angle between the BDT unit and 2‐ethylhexyl thiophene.^[^ ^38^ ^]^ Furthermore, PM6, with the fluorine substituent, exhibits an identical π–π stacking distance to PBDB‐T, suggesting a negligible impact on molecular π–π stacking upon introducing fluorine atoms on the thiophene ring. Interestingly, however, the PM6 shows a much higher intensity of the π–π stacking peak (*q_z_* =  1.66 Å^−1^) as shown in Figure 3d (inset), indicating an additional degree of ordering when fluorine atoms are introduced, which is consistent with the effect of fluorination reported by others.^[^ ^30^, ^32^, ^33^, ^36^ ^]^

**Figure 4:**
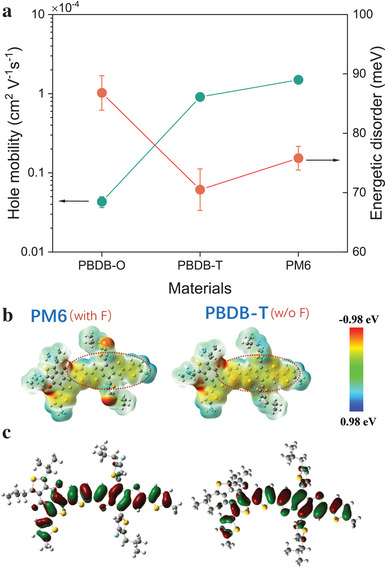
a). Hole mobility and energetic disorder were extracted from the temperature‐dependent *JV* curves for pristine PBDB‐O, PBDB‐T, and PM6, respectively. b). Electrostatic potential distribution and c). the corresponding HOMO calculated by DFT for PBDB‐T (without fluorine) and PM6 (with fluorine).

To better understand the correlation between polymer structure and charge and energy transport properties, we determined the hole mobility and energetic disorder (σ_*DOS*_) for all three pristine CPs through temperature‐dependent space charge limited current–voltage (SCLC *JV*) characteristics using the software reported in ref. [39] Although SCLC requires undoped material, it is the preferred technique to determine the disorder in the part of the DOS that is relevant for charge transport. Importantly, it is commonly assumed that energetic disorder is an additive effect, and in consequence the absolute values of σ_*DOS*_ might change upon doping, leaving the relative trends, in the first order, unaffected. As shown in **Figure** **4**a, the hole mobility of PBDB‐T increases ≈21‐fold, reaching 9.09 ± 0.22 × 10^−5^ cm^2^ V^−1^ s^−1^ upon extending the 2D conjugated structure. This enhancement is attributed to the improved planarity and ordering of the PBDB‐T compared to the PBDB‐O structure, as confirmed by the GIWAXS results discussed earlier. In line with this notion, the energetic disorder of PBDB‐O decreases sharply from 86.8 ± 2.9 to 70.5 ± 3.5 meV for PBDB‐T, which rationalizes the concomitant drop in the Seebeck coefficient.

The effect of fluorination is more complicated and cannot be attributed to a single factor alone: PM6 exhibits higher mobility than PBDB‐T, which one might attribute to the increased molecular ordering via fluorine substitution. However, PM6 surprisingly shows a similar or even larger σ_*DOS*_ of approximately 75.8 ± 2.0 meV. While this is qualitatively consistent with the increased *S* of PM6 as compared to PBDB‐T, it is not consistent with a constant or even increased mobility, as explained in the introduction. This conundrum can be solved by realizing that the mobility is not only proportional to the exponential (Boltzmann) factor, but also contains a prefactor that contains an overlap integral or, equivalently, an attempt‐to‐hop frequency (ν_0_). We attribute (the dominant contribution to) the observed changes in μ and *S* upon fluorination to the combined effects of an increase in σ_*DOS*_ and in ν_0_. Although the latter cannot be measured independently, this would be a logical consequence of the additional degree of ordering when fluorine atoms are introduced. An additional factor contributing to conductivity (but less to mobility) increases would be a more effective dopant intercalation in going from PBDB‐O to PBDB‐T and PM6, which would be consistent with the increasing lamellar spacing in Figure 3f, and could lead to a higher concentration of dopants incorporated in the film at equal dopant concentration in solution. We attribute the ≈40‐fold (at intermediate doping concentrations) conductivity increase between PBDB‐O and PBDB‐T, while the mobility only increases ≈20‐fold to this effect. Later in the paper, we will delve deeper into the cause of high σ_*DOS*_ in fluorinated PM6.

Summarizing the analysis of the GIWAXS and temperature‐dependent *JV* data, we attribute the significant increase in conductivity of PBDB‐T with respect to PBDB‐O to the improvement in the degree of ordering by the planarizing effect of the additional thiophene unit, with a minor (≈2×) contribution from an enhanced doping efficiency at low and intermediate concentrations. The further improvement in power factor in going from PBDB‐T to PM6 is attributed to a further improvement in π–π stacking, in combination with an enhanced energetic disorder, both induced by the fluorination of the CP.

To substantiate the intuition that fluorine substitution can induce localized charges on the backbone, the Coulomb potentials of which then might improve packing while simultaneously increasing the energetic disorder in PM6, we investigated the electrostatic potential surrounding the polymers with and without fluorine. This was achieved by carrying out density functional theory (DFT) calculations at the B3LYP/6‐311G level of theory in a vacuum using the Gaussian 16 program package. As shown in Figure 4b, the electrostatic potential is observed to be almost evenly distributed throughout the backbone of PBDB‐T, except for the modulation caused by the double oxygen in the BDD acceptor block that is present in both polymers. Upon the introduction of fluorine on the thiophene ring of PBDB‐T, we observed a localization of charge density on the side chains of the BDT unit, leading to a disruption in the uniform distribution over the polymer backbone. This suggests that indeed the presence of fluorine may be responsible for the increased energetic disorder observed in PM6, while the induced electrostatic moments may contribute to a stronger intermolecular interaction, promoting packing.

**Figure 5:**
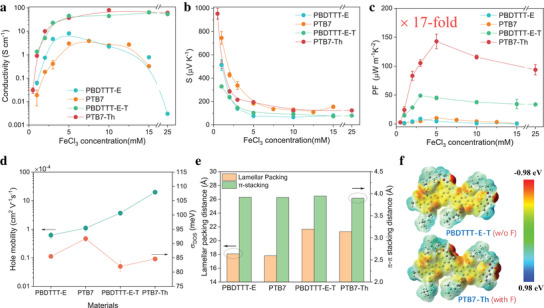
Thermoelectric characteristics of doped CPs as a function of FeCl_3_ concentration for a) Conductivity (𝜎), b) Seebeck coefficient (*S*), and c) power factor (*PF*). d) the hole mobility and energetic disorder extracted from the temperature‐dependent JV curves, e) lamellar packing distance (100) and π–π stacking distance (010) for pristine PBDTTT‐E, PTB7, PBDTTT‐E‐T, and PTB7‐Th, respectively. f) the electrostatic potential distribution calculated by DFT for PBDTTT‐E‐T (without fluorine) and PTB7‐Th (with fluorine).

To investigate the universality of our proposed two‐step design rule, we investigate the thermoelectric properties of the PTB7‐Th series of materials, including PBDTTT‐E, PTB7, PBDTTT‐E‐T, and PTB7‐Th, which follows the same line of design, with the added element that PTB7 is fluorinated while lacking the planarizing alkyl‐thiophene groups on the BDT donor block. The measured conductivity, Seebeck coefficient, and *PF* are shown in **Figure** **5**a–c. Ignoring the PTB7 for now, which does not have an equivalent in the PM6‐series, we note that the conductivity and Seebeck coefficient of the PTB7‐Th series follow similar trends as observed in the PM6 series. As compared to the PBDTTT‐E base material, the conductivity of PBDTTT‐E‐T (planarization) and PTB7‐Th (fluorination) shows a, in this case similar, remarkable improvement by a factor ≈10, attributed to the extended 2D conjugation and, in the case of PTB7‐Th, the additional joint effects of enhanced packing and increased electrostatic disorder. For the Seebeck coefficient, it is evident that the fluorine substitution in PTB7‐Th again leads to higher values, reaching 194.6 ± 7.6 µV K^−1^ at maximum *PF*, compared to the corresponding value of 146.0 ± 6.8 µV K^−1^ of PBDTTT‐E‐T. As seen in Figure 5d,e, the measured hole mobilities, energetic disorder, and the GIWAXS data also demonstrate similar trends to those observed in the PM6 series and, hence, can be connected to the thermoelectric performance indicators (σ, *S*) by the same arguments. In particular, we note again the mobility increase between PBDTTT‐E‐T and PTB7‐Th, despite a modest increase in energetic disorder (σ_*DOS*_) (**Table** **1**).

**Table 1:** Summary of the extracted GIWAXS data, hole mobility, and energetic disorder for all CPs studied in this work.

Materials	q_100_ [nm^−1^]	d_100_ [Å]	q_010_ [nm^−1^]	d_010_ [Å]	Mobility^a)^ [cm^2^ V^−1^ s^−1^]	σ_*DOS*_ [meV]
PBDB‐O	3.50	17.95	16.88	3.72	(4.31 ± 0.68) × 10^−6^	86.8 ± 2.9
PBDB‐T	3.34	18.81	16.58	3.79	(9.09 ± 0.22) × 10^−5^	70.5 ± 3.5
PM6	3.30	19.04	16.58	3.79	(1.50 ± 0.14) × 10^−4^	75.8 ± 2.0
PBDTTT‐E	3.50	17.95	16.00	3.93	(6.18 ± 1.40) × 10^−5^	85.5 ± 0.3
PTB7	3.58	17.83	16.20	3.92	(1.10 ± 0.24) × 10^−4^	91.7 ± 0.9
PBDTTT‐E‐T	3.00	20.94	16.00	3.93	(3.68 ± 0.65) × 10^−4^	82.0 ± 1.9
PTB7‐Th	3.00	20.94	16.23	3.87	(1.99 ± 0.13) × 10^−3^	84.6 ± 0.7

^a)^ The measured mobility at room temperature.

We note that the balance of the beneficial and adverse effects of fluorination on conductivity is overall slightly more positive for PTB7‐Th than for PM6. This difference may be due to the influence of the position of the fluorine atom, sitting either on the D (in PM6) or the A (in PTB7‐Th) unit, of the CP backbone. In the literature, as listed in Table S2 (Supporting Information), a similar variability in the effect of fluorination on conductivity is observed. Likewise, the increase in *S* upon the inclusion of fluorine has been observed in the literature, cf. Table S2 (Supporting Information). Interestingly, in comparison to the slow increase in conductivity observed in PM6, the conductivity of PTB7‐Th rapidly surpasses 10 S cm^−1^, reaching levels comparable to those of PBDTTT‐E‐T under low FeCl_3_ concentration. This may tentatively be attributed to p‐type doping occurring primarily between the FeCl_3_ dopant and the BDT donor unit. Then, the negative potential at the fluorine atom might push away the dopant anion from the BDT unit in the case of PM6 while it may at least negligibly push it away from the BDT unit in case the fluorine is on the acceptor unit, as in PTB7‐Th. We are aware other factors may additionally contribute, which is beyond the scope of the present work. However, in view of the similarities observed between the PM6 series and PTB7‐Th series, our findings suggest that fluorine substitution represents a universal approach to enhance the Seebeck coefficient, irrespective of whether fluorine is introduced on the donor or acceptor unit in D‐A copolymers. Furthermore, to assess the “universal” effect of fluorination, we have retrieved literature on the impact of fluorination on Seebeck coefficients. It is noted that the introduction of fluorine atoms always enhances the Seebeck coefficient, at least to some degree, for p‐type thermoelectric materials (see Table S2, Supporting Information). However, the situation differs for n‐type materials due to the challenges posed by n‐type doping. We attribute the effect of fluorination in n‐type materials to the downward shift of the LUMO level, which significantly enhances the doping efficiency and reduces the gap between the Fermi energy and transport energy. This results in a marked increase in electrical conductivity, but also a distinct decrease in the Seebeck coefficient (see Table S3, Supporting Information).

The observations for PTB7 fit well in the trends above and as such further confirm the 2‐step design protocol. As for the pair PBDTTT‐E – PTB7, fluorination of PBDTTT‐E to yield PTB7 gives rise to a modest increase in mobility despite an, in this case, more significant, increase in disorder. In turn, the increase in σ_*DOS*_ can one on one be translated to an enhancement in *S* as per the arguments in the introduction. We attribute the similar or even lower conductivity of PTB7 as compared to PBDTTT‐E to a slightly less efficient dopant intercalation, as inferred from the reduced lamellar packing distance. More importantly, lacking the 2D planarizing alkyl‐thiophene groups, the conductivity of PBDTTT‐E and PTB7 at maximum power point falls significantly below that of PBDTTT‐E‐T and PTB7‐Th.

Summarizing our findings on the PTB7‐Th series, through the implementation of the two‐step design rule, we achieve an optimal *PF* exceeding 155 (142.7 ± 12.7) µW m^−1^ K^−2^ for PTB7‐Th, representing a remarkable 17‐fold enhancement compared to its reference counterpart, PBDTTT‐E, which exhibits a PF 8.88 ± 0.23 µW m^−1^ K^−2^.

Finally, we want to emphasize that the relationship between the Seebeck coefficient and energetic disorder is complex, and one should not expect monotonous trends across significantly different materials. While σ_*DOS*_ plays a role in influencing *S*, other factors such as structural order can impact the dopant distribution over amorphous and crystalline phases. Additionally, variations in dopant orientation relative to the polymer backbone, along with differences in dielectric constant, can affect charge transfer efficiency and the position of the *E_F_*. Therefore, comparisons should be made between polymers with as similar characteristics as possible. In pairs such as PBDB‐T versus PM6, PBDTTT‐E versus PTB7, and PBDTTT‐E‐T versus PTB7‐Th, the correlation between σ_*DOS*_ and *S* is consistent. At the same time, we note that the global picture is more complex, highlighting the importance of secondary factors and the need for further study.

We want to briefly address the factor of the dielectric constant impacting the conductivity and Seebeck coefficient. Upadhyaya et al. have suggested that increasing the dielectric constant can enhance thermoelectric properties, ascribed to the charge screening effect in high dielectric constant systems,^[^ ^40^ ^]^ which alleviates Coulomb interactions induced by dopants. It has been reported that introducing and/or increasing the fluorine element can enhance the dielectric constant of materials.^[^ ^32^, ^33^, ^35^, ^41^ ^]^ To investigate the relevance of this effect for our approach, we measured the dielectric constant for all polymers used in this study, following a device structure of ITO/active layer/Ag. The results are presented in Figure S19 (Supporting Information). For the PM6 series, the dielectric constant gradually increases with the subsequent steps in the two‐step design rule, which might support a causal relation. However, in the PTB7‐Th series, the trend is not consistent. In addition, we note that a systematic increase in dielectric constant might indeed be expected to cause a reduction in electrostatic disorder, which would promote the conductivity. However, this would also reduce the Seebeck coefficient, in stark contrast to our observations for fluorinated polymers.

It is worth noting that the measurement of the dielectric constant strongly depends on factors such as film geometry, preferential alignment in the edge‐ or face‐on direction, film anisotropy, and so on. These factors may contribute to the variability observed in the dielectric constant measurements. We may explore the impact of dielectric constant further in future investigations, but this is beyond the scope of the current study.

## 3 Conclusion

In summary, we propose a practical two‐step design rule capable of simultaneously achieving high conductivity and Seebeck coefficients for conjugated polymers. The planarization due to the extension of the backbone in 2D enhances conductivity by improving molecular packing order and charge mobility, while potentially allowing for increased dopant intercalation due to the larger lamellar packing distance. Regardless of its position on the backbone, fluorine substitution proves to be an effective method for increasing *S*, while having only a modest or even negligible adverse effect on the conductivity. This is attributed to a modest increase in energetic disorder σ_*DOS*_, resulting from the strong electron affinity of fluorine, in combination with a further improvement of the π–π stacking. Based on this two‐step design strategy, we achieve an optimal *PF* ≈88 (85.2 ± 3.4) µW m^−1^ K^−2^, along with *S* ≈ 123.5 (123.5 ± 0.5) µV K^−1^, in the PM6 series, which represents a 32‐fold enhancement compared to the reference PBDB‐O material. Likewise, in the PTB7‐Th series, we achieve a maximum *PF* ≈155 (142.7 ± 12.7) µW m^−1^ K^−2^, coupled with *S* ≈202 (194.6 ± 7.6) µV K^−1^, representing a ≈17‐fold improvement compared to the reference PBDTTT‐E. To conclude, the two‐step design rule emerges as a promising design strategy to overcome the coupling between σ and *S* and to achieve high conductivity and high Seebeck coefficients in conjugated polymers.

## 4 Experimental Section

### Materials

The polymers PM6, PBDTTT‐E, PBDTTT‐E‐T, PTB7, and PTB7‐Th were purchased from Solarmer Materials Inc. Polymers PBDB‐O, and PBDB‐T were synthesized and provided by Hou's group.^[^ ^42^ ^]^ FeCl_3_ (≥ 99.9%) was purchased from Aladdin Biochemical Technology Co., LTD. The solvent acetonitrile (AR, ≥ 99.0%) was purchased from Sinopharm Chemical Reagent Co., Ltd., and chlorobenzene (AR, ≥ 99.5%) was purchased from Shanghai Titan Scientific Co Ltd. All materials were used as received.

### Device Fabrication

The glass substrates with patterned indium tin oxide (ITO) electrodes were sonicated successively with soapsuds, deionized water, acetone, ethyl alcohol, and iso‐propanol and dried with nitrogen flow. The polymer solution of PBDB‐O (5 mg mL^−1^ in chlorobenzene), PBDB‐T, PM6, PBDTTT‐E, PBDTTT‐E‐T, PTB7 and PTB7‐Th (10 mg mL^−1^ in chlorobenzene) were spin‐coated at speed of 2000 rpm min^−1^ for 45 s, 3000 rpm min^−1^ for 25 s and then annealed at 160 °C for 20 min. For doped polymer thin films, the pre‐annealed films were immersed in FeCl_3_/acetonitrile solution of various concentrations for 60 s followed by spinning off the remaining solution. The doped films were washed with acetonitrile once and then annealed at 60 °C for 10 min to remove the residual solvent. The film thickness is ≈36–72 nm measured with a Dektak surface profilometer.

### Hole‐Only Device Fabrication and Temperature Dependent JV Measurement

The devices were fabricated with a structure ITO/PEDOT:PSS/Polymer/MoO_3_/Ag. PEDOT:PSS was filtered through a 0.22 µm poly(tetrafluoroethylene) (PTFE) and then spin‐coated on top of ITO with 2000 rpm min^−1^ for 40 s, followed by thermal annealing on a hot plate with 150 °C for 20 min in air. The layer of polymer (PBDB‐O 5 mg mL^−1^ and others 20 mg mL^−1^ in chlorobenzene) was spin‐coated with 1000 rpm min^−1^ for 60 s. Subsequently, the MoO_3_ (10 nm) and Ag (100 nm) electrodes were deposited through a shadow mask under a pressure of ≈5 ×  10^−4^ Pa. The active area was 8 mm^2^ between the two electrodes. The hole‐only devices were measured in a custom light‐blocking chamber maintained under a vacuum environment of less than 0.1 Pa. Temperature control was achieved using liquid nitrogen and a heater. *JV* curves were measured using a Keithley 2450 after allowing 30 min of stabilization at each temperature gradient.

### Conductivity and Seebeck Coefficient Measurement

All measurements were performed immediately after device fabrication in a glove box under a Nitrogen atmosphere. Room temperature electrical characterizations were performed in a glove box under a Nitrogen atmosphere. Current–voltage characteristics were obtained between −50 and 50 mV. Conductivities were calculated according to σ  =  *j*/*F*, where *j* and *F* are the current density and electric field, respectively. The thermopower was obtained by applying a linear temperature gradient of various magnitudes along the sample and recording the shift of the *jV* characteristics at the end of a settling time of ≈400 s. From the change of the thermovoltage Δ*V* as a function of the temperature difference Δ*T* between the contacts, the thermopower *S* was calculated as *S*  =  Δ*V*/Δ*T*. Each doping concentration was tested on approximately 6–8 devices, across 3–4 batches, on different days.

### Other Characterization

UV–vis–NIR spectra were measured by Lambda 750. GIWAXS profiles were recorded at beamline BL14B1 of the Shanghai Synchrotron Radiation Facility (SSRF) at a wavelength of 1.2398 Å. AFM height images were acquired using tapping‐mode atomic force microscopy (Dimension Edge, Bruker). The dielectric constant was measured using an impedance analyzer.

### Statistical Analysis

All experiments were conducted at least three times with similar results. Data of thermoelectric measurements, hole mobility, and disorder fitting results were reported as mean values ± standard deviations, as shown in Figures 2, 4 and 5. Statistical tests were two‐sided if not mentioned otherwise. Statistical analysis was conducted using OriginPro software.

## Conflict of Interest

The authors declare no conflict of interest.

## Supporting information



Supporting Information

## Data Availability

The data that support the findings of this study are available from the corresponding author upon reasonable request.
