# Supplementary material for: Two‐Step Design Rule for Simultaneously High Conductivity and Seebeck Coefficient in Conjugated Polymer‐Based Thermoelectrics
Source: Adv Sci (Weinh). 2024 Nov 8;12(1):2409382. doi: 10.1002/advs.202409382 (PMC11714185; doi:10.1002/advs.202409382)
Supplement: Supplementary file 1 — Supporting Information [file ADVS-12-2409382-s001.pdf]

ADVANCED SCIENCE
Open Access

Supporting Information

for *Adv. Sci.*, DOI 10.1002/advs.202409382

Two-Step Design Rule for Simultaneously High Conductivity and Seebeck Coefficient in Conjugated Polymer-Based Thermoelectrics

Zelong Li, Wei Fu, Dorothea Scheunemann, Xiaoran Wei, Maximilian Litterst, Priya Mariam Viji, Yong Cui, Jianhui Hou, Junhui Tang, Ziqi Liang, Zehua Qu, Martijn Kemerink*, Ruiqian Guo and Guangzheng Zuo**

Supporting Information

Two-Step Design Rule for Simultaneously High Conductivity and Seebeck Coefficient in Conjugated Polymer-Based Thermoelectrics

Zelong Li[1], Wei Fu[1], Dorothea Scheunemann[2], Xiaoran Wei[1], Maximilian Litterst[2], Priya Viji[2], Yong Cui[3], Jianhui Hou[3], Junhui Tang[4], Ziqi Liang[4], Zehua Qu[5], Martijn Kemerink[2]*, Ruiqian Guo[1], Guangzheng Zuo[1]**

Contents

S1 – UV–vis–NIR absorption spectra 2
S2 – TE characteristics of polymers studied in this paper 4
S3 – AFM images of polymers studied in this paper 7
S4 – 2D-GIWAXS profiles and the extracted data 9
S5 – Temperature dependent SCLC JV curves for hole-only devices 10
S6 – DFT calculation for electrostatic potential surfaces and energy level 12
S7 – Measured dielectric constants for all used materials 13
S8 – Summary of TE properties for CPs with (without) fluorine 14
S9 –Summary of mobilities for CPs with 1D and 2D structure in literatures 17
References 19

S1 – UV–vis–NIR absorption spectra

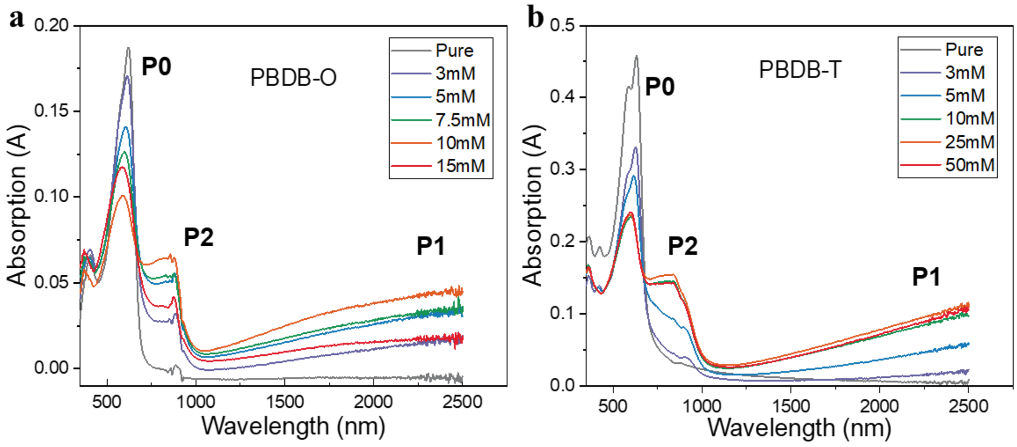

Figure S1. UV–vis–NIR absorption spectra of pristine and doped films for PM6 series studied in this work.

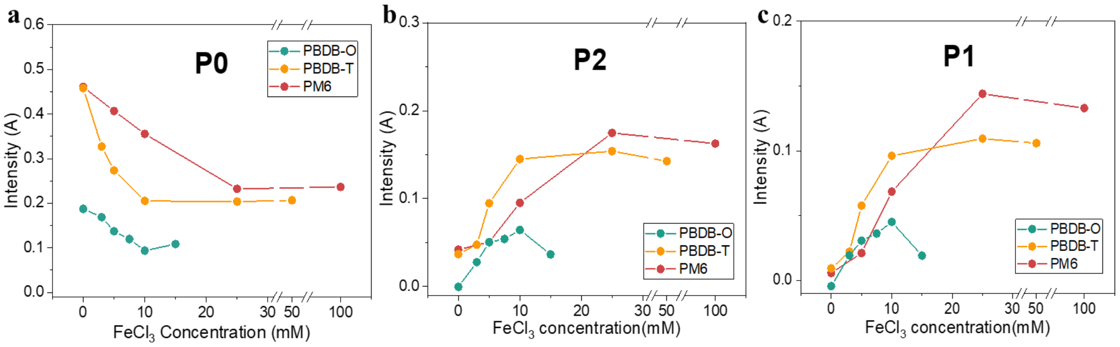

Figure S2. Absorption intensity of P0, P1and P2 in Figure 2d and Figure S1 as a function of dopant concentration for PM6 series.

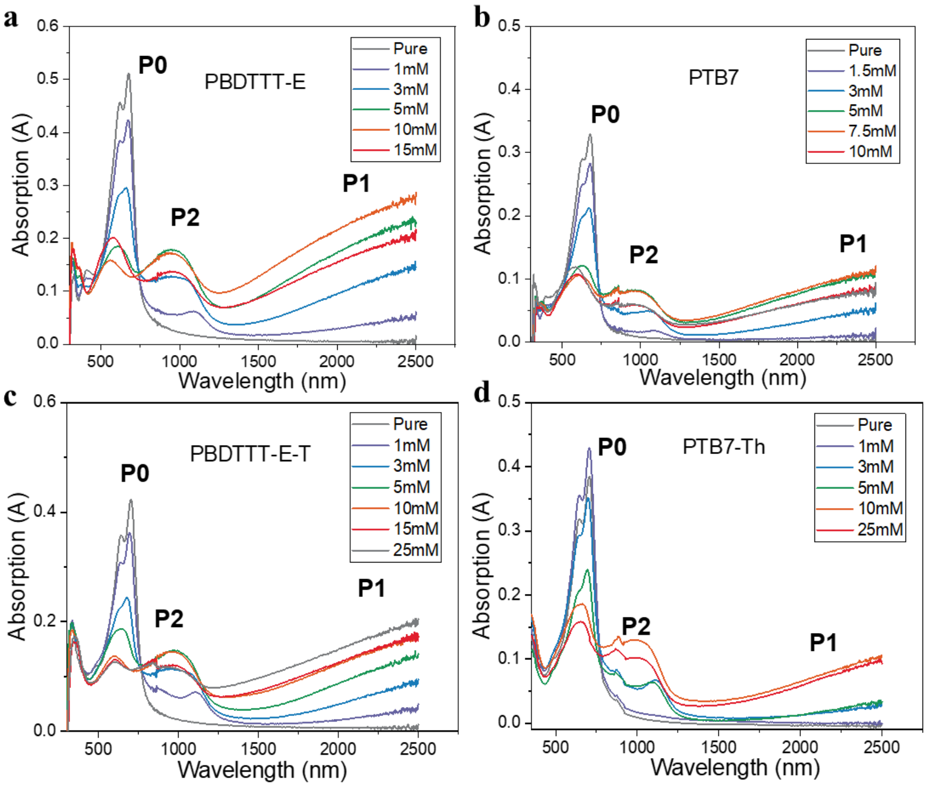

Figure S3. UV–vis–NIR absorption spectra of pristine and doped films for PTB7-Th series studied in this work.

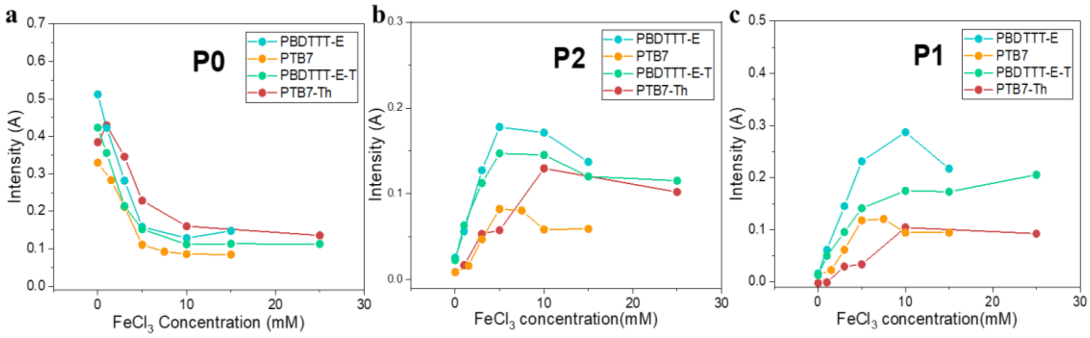

Figure S4. Absorption intensity of P0, P1 and P2 in Figure S3 as a function of dopant concentration for PTB7-Th series.

S2 – TE characteristics of polymers studied in this paper

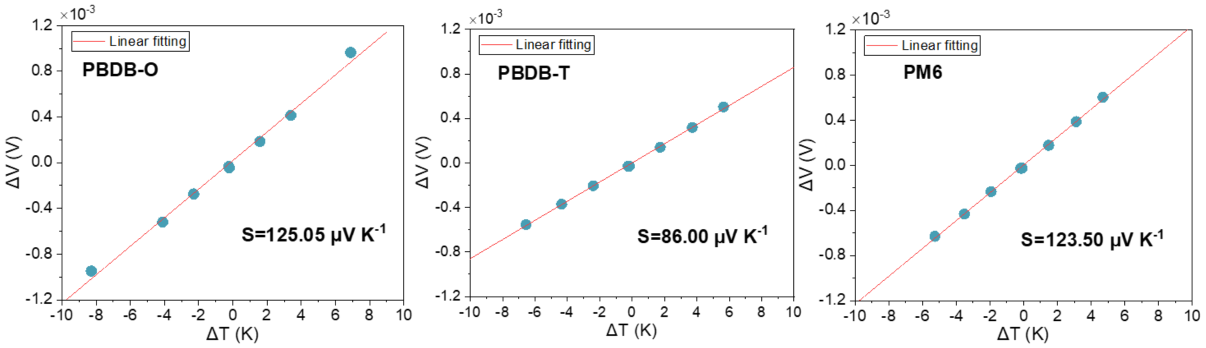

Figure S5. The measured Seebeck coefficient at optimal power factor for PM6 series.

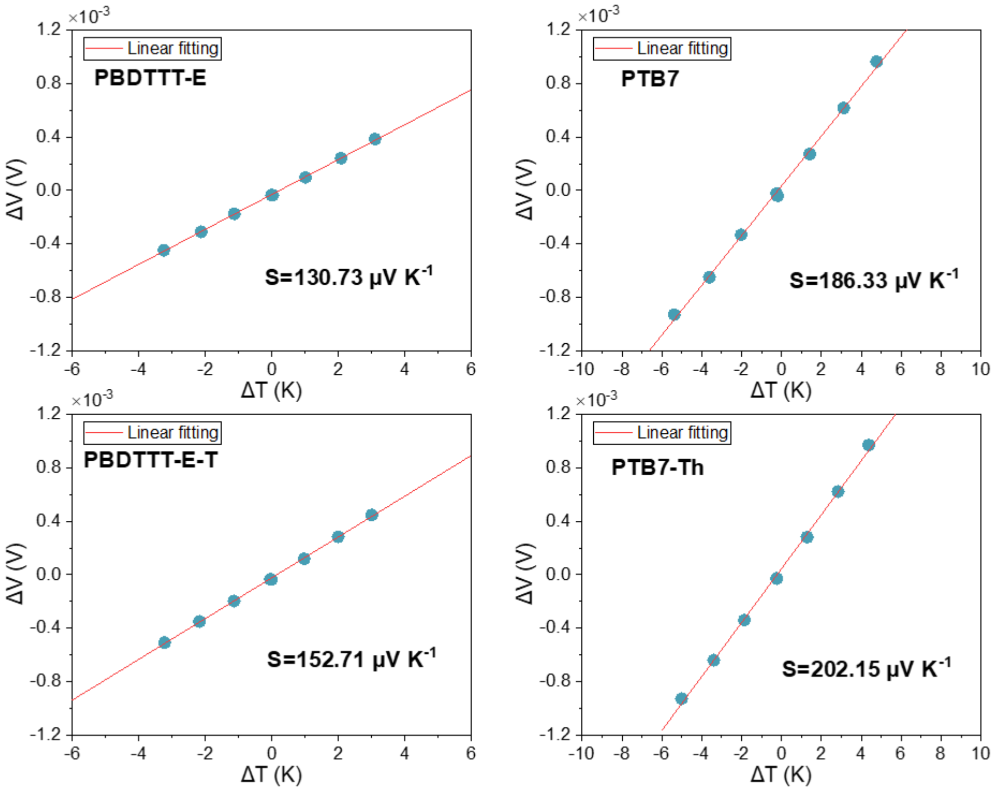

Figure S6. The measured Seebeck coefficient at optimal power factor for PTB7-Th series.

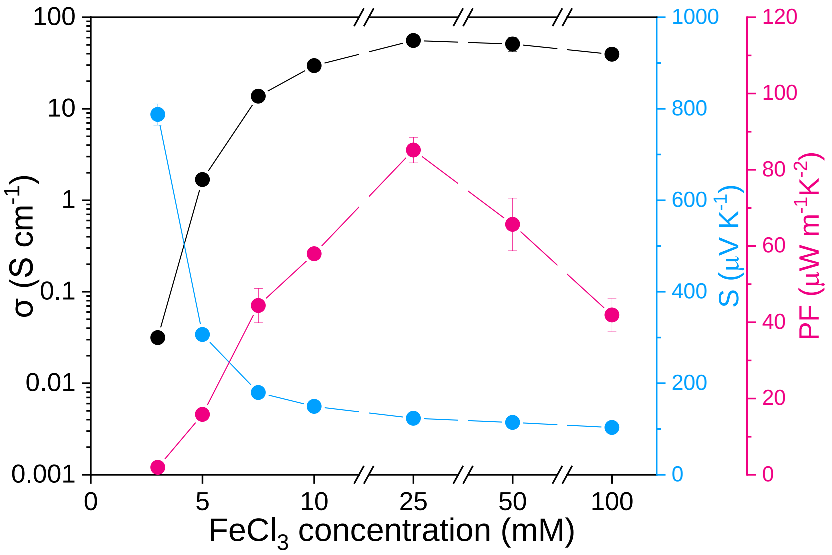

Figure S7. Thermoelectric characteristics of PM6 as a function of $FeCl_3$ concentration.

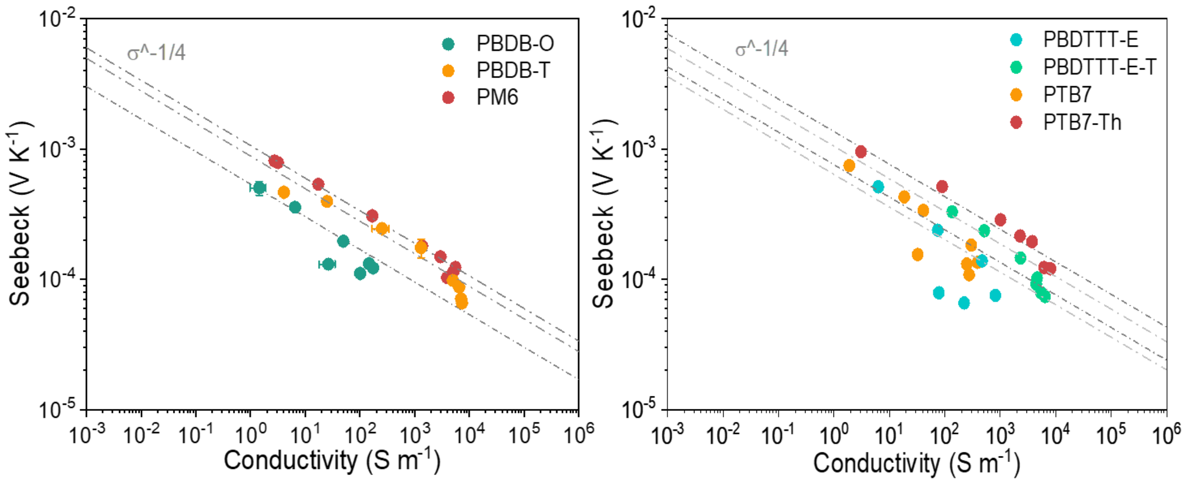

Figure S8. Seebeck coefficient vs. Conductivity for all studied polymers.

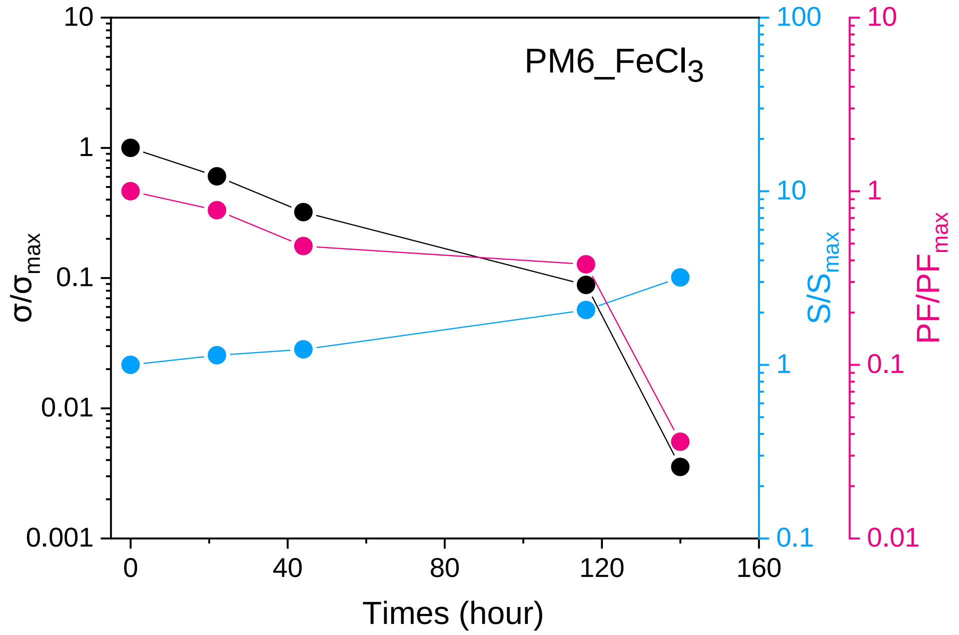

Figure S9. The stability of PM6 doped with 25mM $FeCl_3$ with times.

Table S1. Optimal measured thermoelectric properties of CPs studied in this work.

Materials	Conductivity ($S\ cm^{-1}$)	Seebeck ($\mu V\ K^{-1}$)	PF ($\mu W\ m^{-1}K^{-2}$)
PBDB-O (7.5mM)	1.74 ± 0.04	122.3 ± 2.8	2.60 ± 0.18
PBDB-T(7.5mM)	65.2 ± 2.8	87.0 ± 1.0	49.4 ± 0.9
PM6 (25mM)	55.7 ± 2.4	123.5 ± 0.5	85.2 ± 3.4
PBDTTT-E (3mM)	4.69 ± 0.64	138.4 ± 7.7	8.88 ± 0.23
PTB7 (5mM)	3.00 ± 0.06	182.7 ± 3.7	10.0 ± 0.2
PBDTTT-E-T (3mM)	23.1 ± 1.8	146.0 ± 6.8	48.9 ± 0.7
PTB7-Th (5mM)	37.6 ± 0.4	194.6 ± 7.6	142.7 ± 12.7

S3 – AFM images of polymers studied in this paper

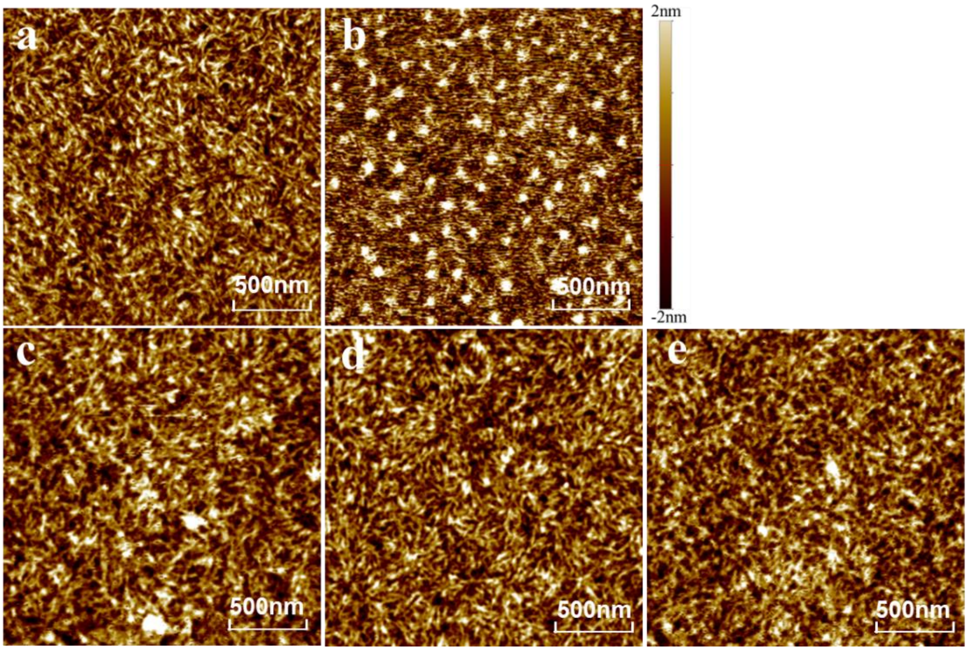

Figure S10. AFM height images of PM6 films with different $FeCl_3$ doping concentration a). pristine, b).5 mM, c).10 mM, d).50 mM, e.)100 mM. All sizes 2 × 2 μm.

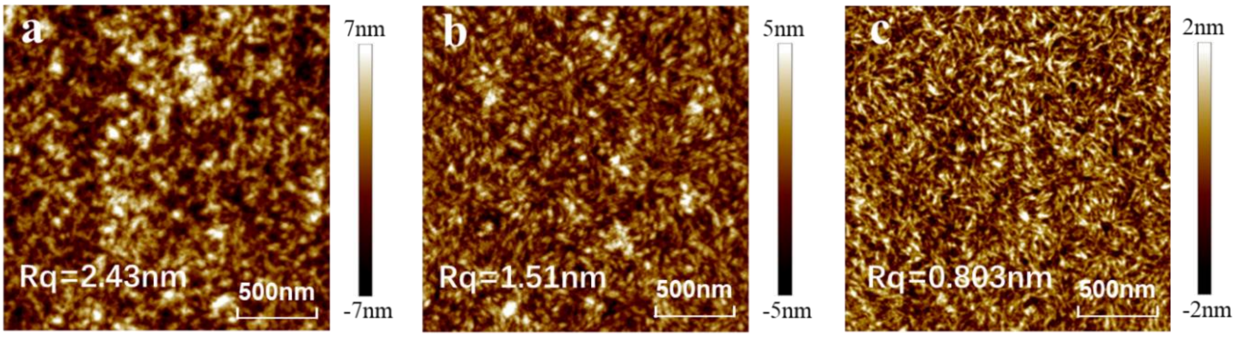

Figure S11. AFM and roughness for pristine a). PBDB-O, b). PBDB-T, c). PM6. All sizes 2 × 2 μm.

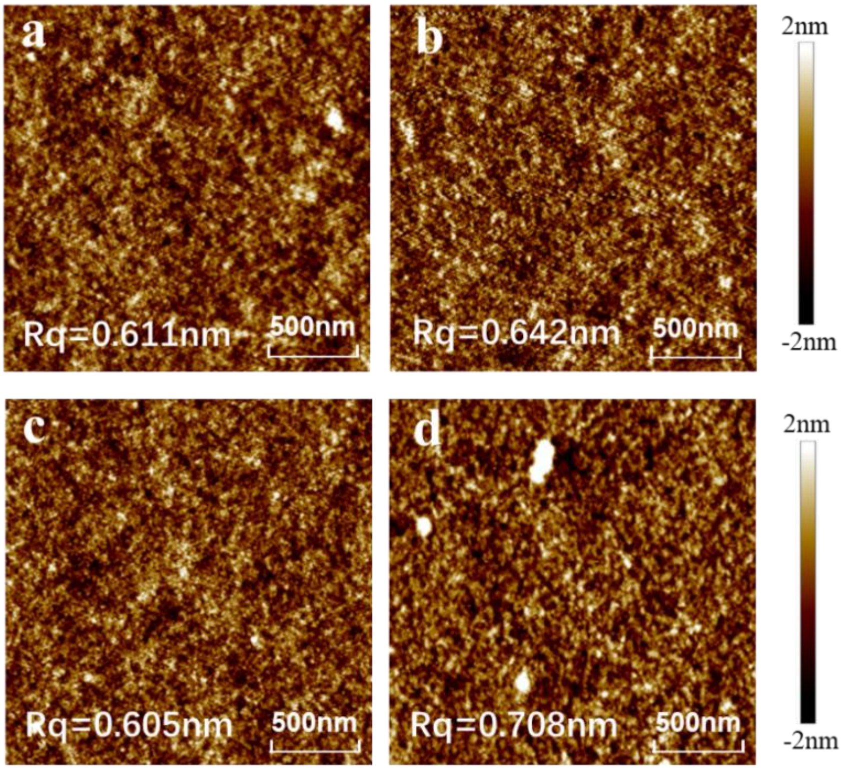

Figure S12. AFM and roughness for pristine a). PBDTTT-E, b). PBDTTT-E-T, c). PTB7, d). PTB7-Th. All sizes 2 × 2 μm.

S4 – 2D-GIWAXS profiles and the extracted data

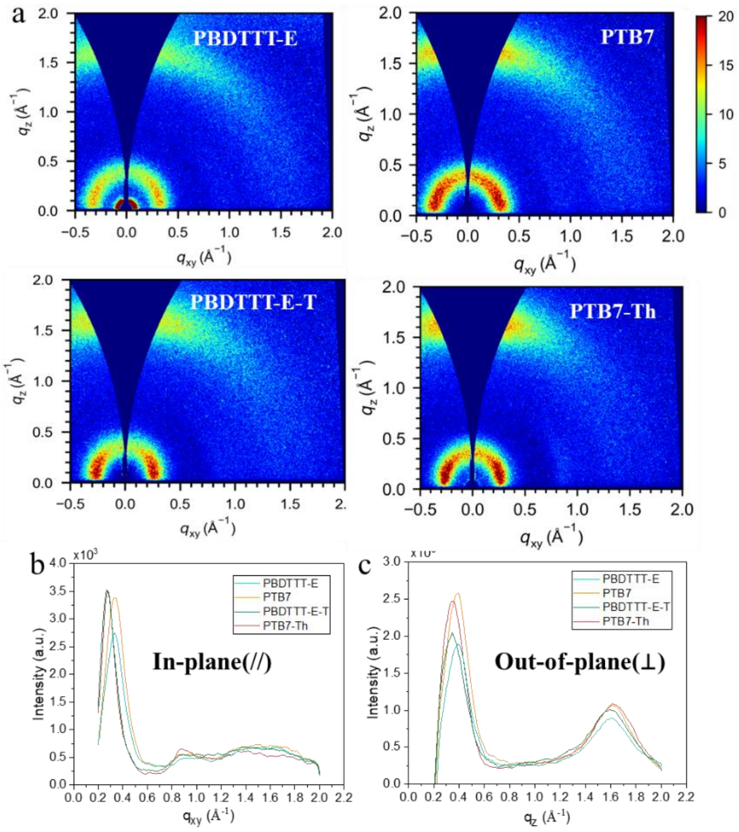

Figure S13. a). 2D-GIWAXS profiles of PTB7, PTB7-Th, PBDTTT-E and PBDTTT-E-T. The scattering profiles b). in-plane (‖), c). out-of-plane (⊥) and the scattering intensity of lamellar packing (100) and pi-packing (010) peak for PTB7 system CPs.

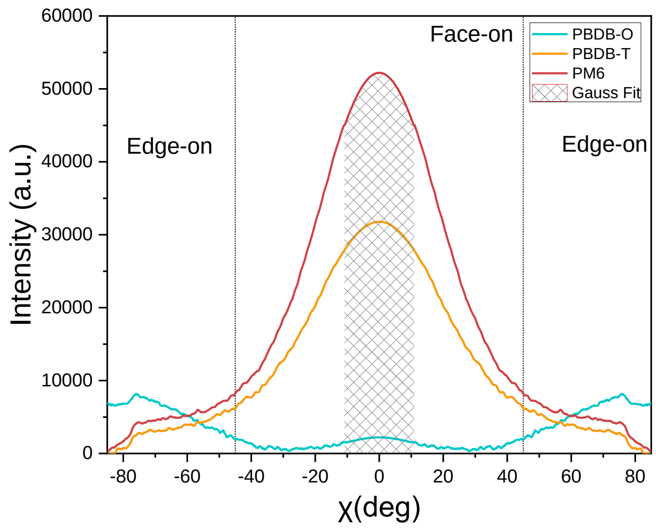

Figure S14. Pole figures extracted from the (010) diffractions for PM6 system polymers.

S5 – Temperature dependent SCLC JV curves for hole-only devices

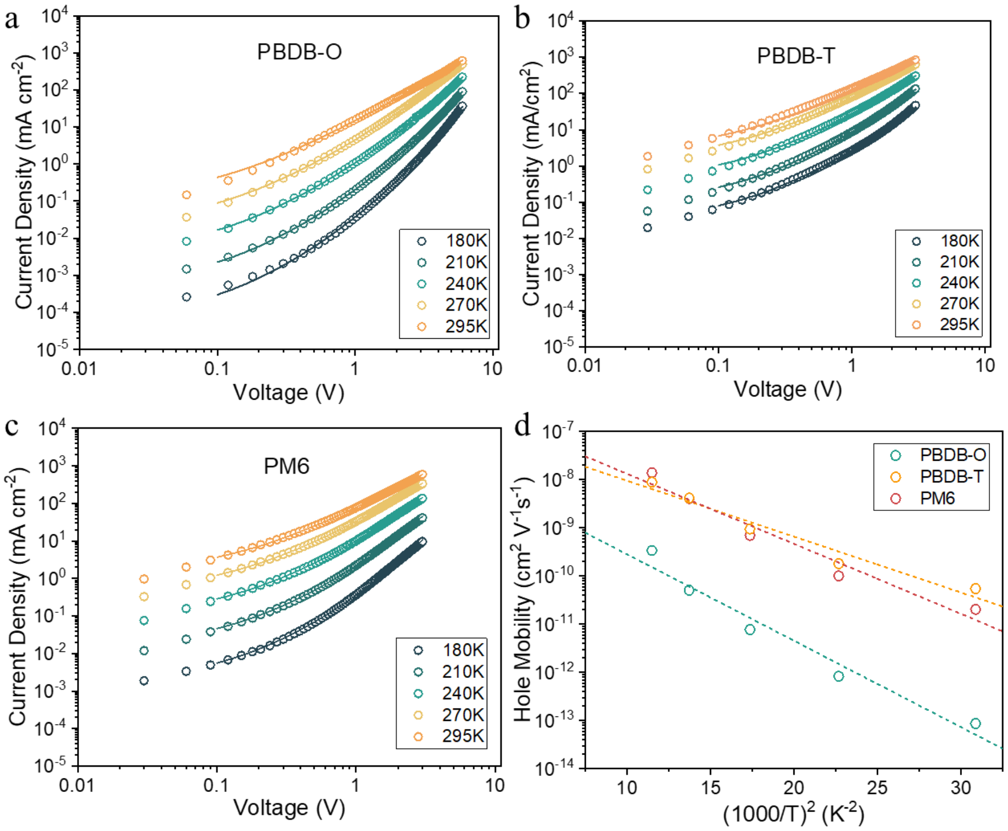

Figure S15. The temperature dependent space charge limited JV curves for pristine a). PBDB-O, b). PBDB-T and c). PM6, and d). The zero-field hole mobility for the corresponding PM6 series of CPs. (The JV fitting model followed the Mott-Gurney + GDM constrained mode [1] in this work)

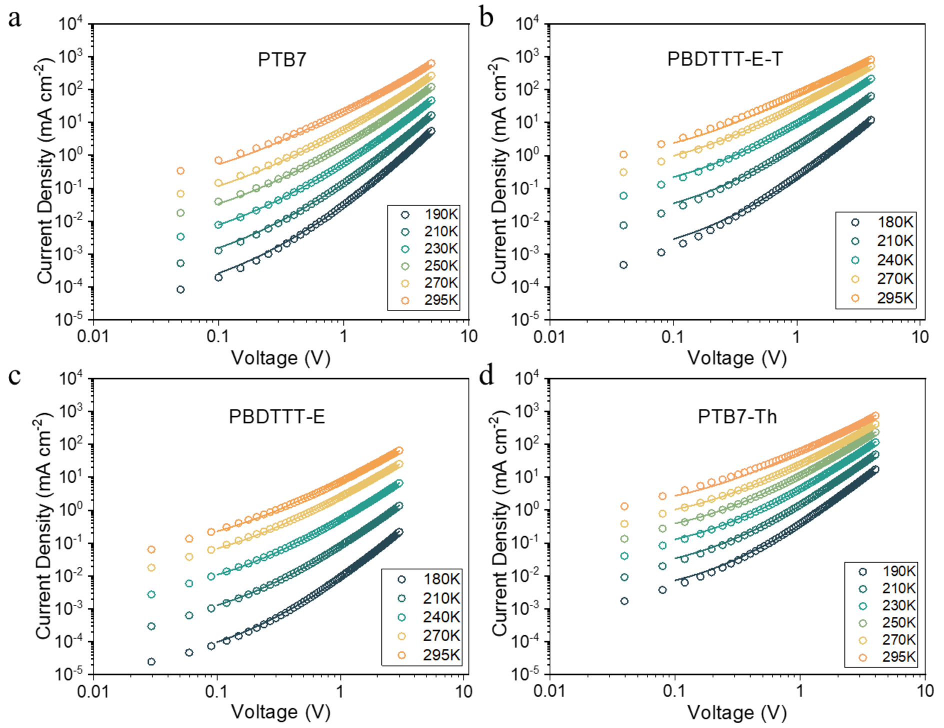

Figure S16. The temperature dependent JV curves for pristine a). PTB7, b). PBDTTT-E-T, c). PBDTTT-E and d). PTB7-Th.

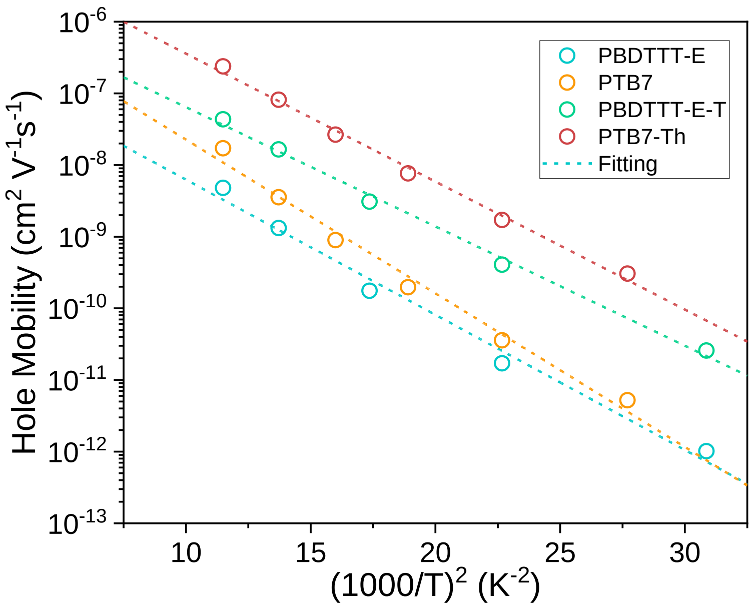

Figure S17. The zero-field hole mobility for the corresponding PTB7 series of CPs in Figure S9.

S6 – DFT calculation for electrostatic potential surfaces and energy level

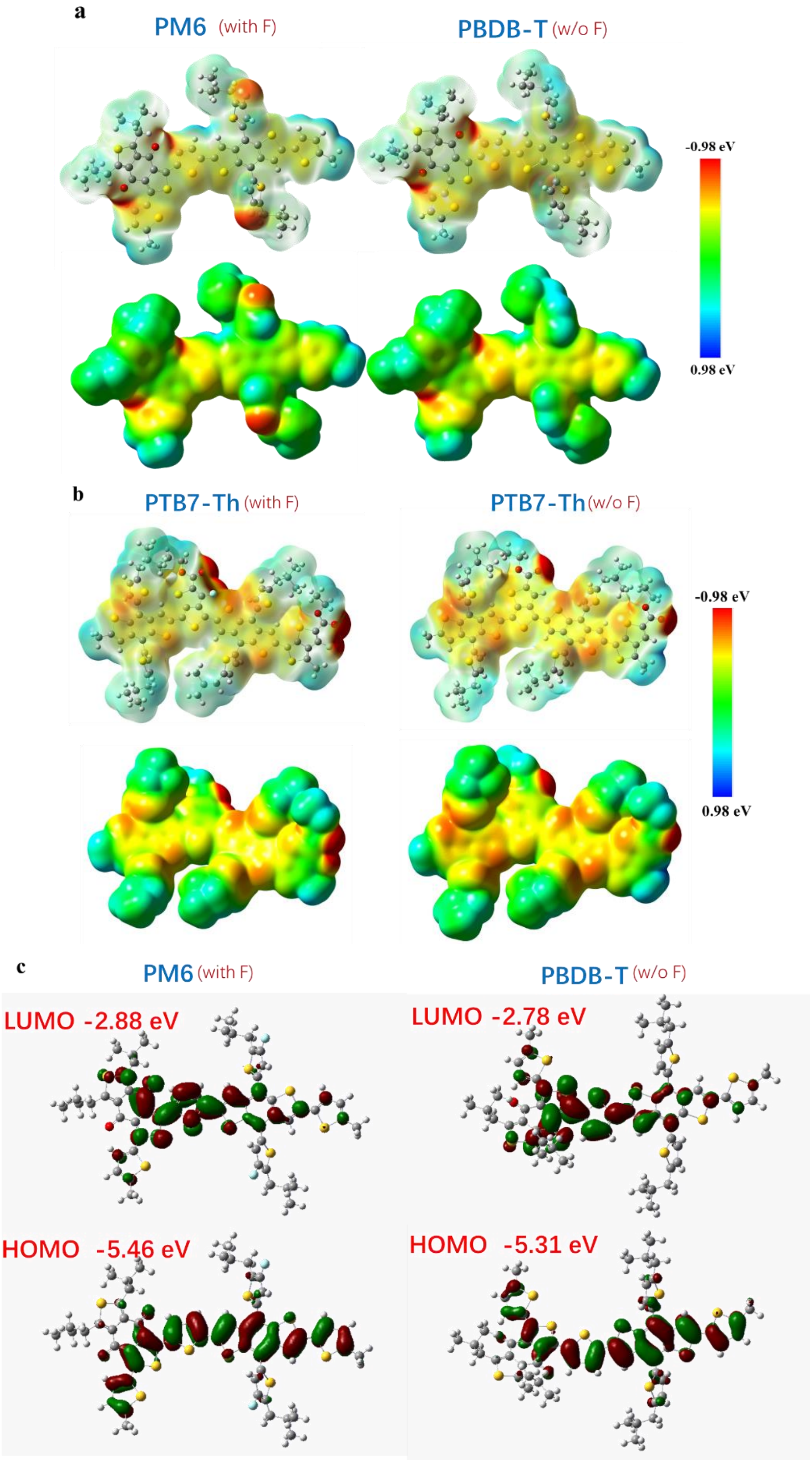

Figure S18. DFT calculation for electrostatic potential surfaces of a). PM6 and PBDB-T, b). PTB7-Th and PTB7-Th, and c). HOMO and LUMO distributions of PM6 and PBDB-T.

S7 – Measured dielectric constants for all used materials

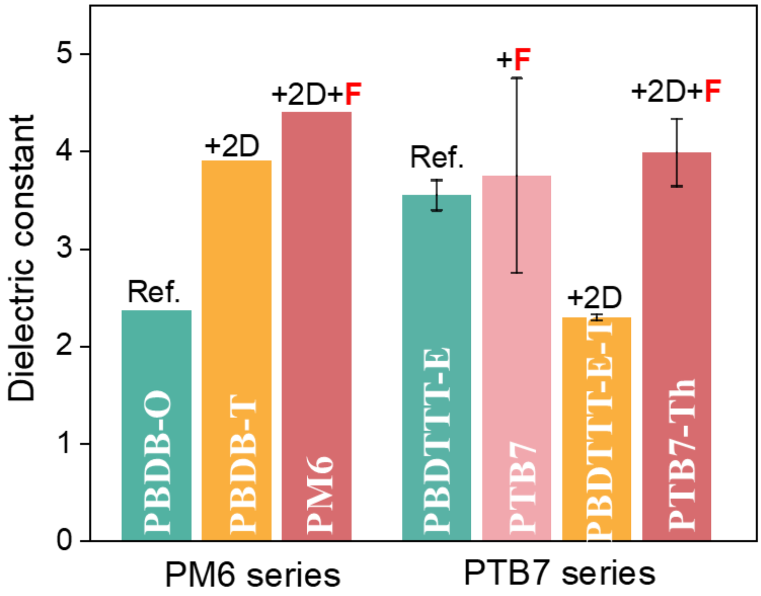

Figure S19. The measured dielectric constant for CPs studied in the work.

S8 – Summary of TE properties for CPs with (without) fluorine

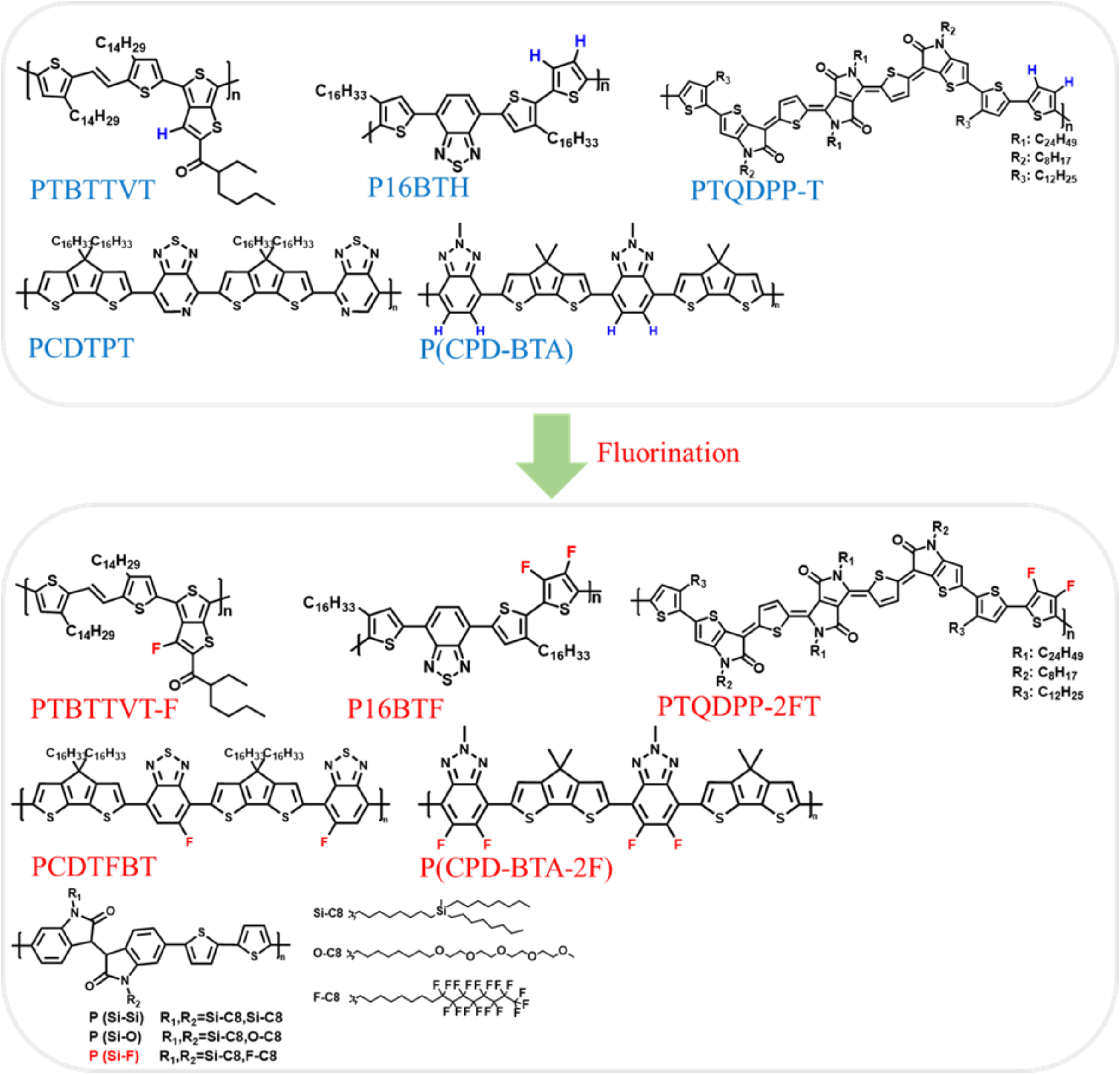

Figure S20. The chemical structure of p-type of CPs with (without) fluorine (F) from literature.

Table S2 Thermoelectric parameters of p-Type polymers with and without fluorine.

Materials	Dopant	σ (S cm^{-1})	S (μV K^{-1})	PF (μW m^{-1}K^{-2})	Reference
PTBTTVT	CuTFSI	180.7	17.7	5.6	Ref.2
**PTBTTVT-F**	CuTFSI	111.1	**25.5**	7.2	Ref.2
PTBTTVT	CN6CP	59.1	85.1	42.8	Ref.2
**PTBTTVT-F**	CN6CP	30.7	**107.5**	35.5	Ref.2
P16BTH	$FeCl_3$	118.7	43.5	22.4	Ref.3
**P16BTF**	$FeCl_3$	47.2	**59.9**	16.9	Ref.3
PTQDPP-T	$FeCl_3$	108.0	66.5	47.8	Ref.4
**PTQDPP-2FT**	$FeCl_3$	168.0	**118.9**	237.5	Ref.4
PCDTPT	F_4TCNQ	5.13	211	21.8	Ref.5
**PCDTFBT**	F_4TCNQ	6.91	**213**	31.5	Ref.5
P(CPD-BTA)	$FeCl_3$	0.43	123	0.65	Ref.6
**P(CPD-BTA-2F)**	$FeCl_3$	4.71	**128**	7.77	Ref.6
P(Si-Si)	$FeCl_3$	10.2	104.9	11.2	Ref.7
P(Si-O)	$FeCl_3$	27.3	92.5	23.4	Ref.7
**P(Si-F)**	$FeCl_3$	3.2	**119.1**	4.5	Ref.7
PBDB-T	$FeCl_3$	68.06	86.0	50.33	**This work**
**PM6**	$FeCl_3$	58.04	**123.5**	88.52	**This work**
PBDTTT-E	$FeCl_3$	5.33	130.7	9.11	**This work**
**PTB7**	$FeCl_3$	2.94	**186.3**	10.21	**This work**
PBDTTT-E-T	$FeCl_3$	21.26	152.7	49.58	**This work**
**PTB7-Th**	$FeCl_3$	38.03	**202.15**	155.41	**This work**

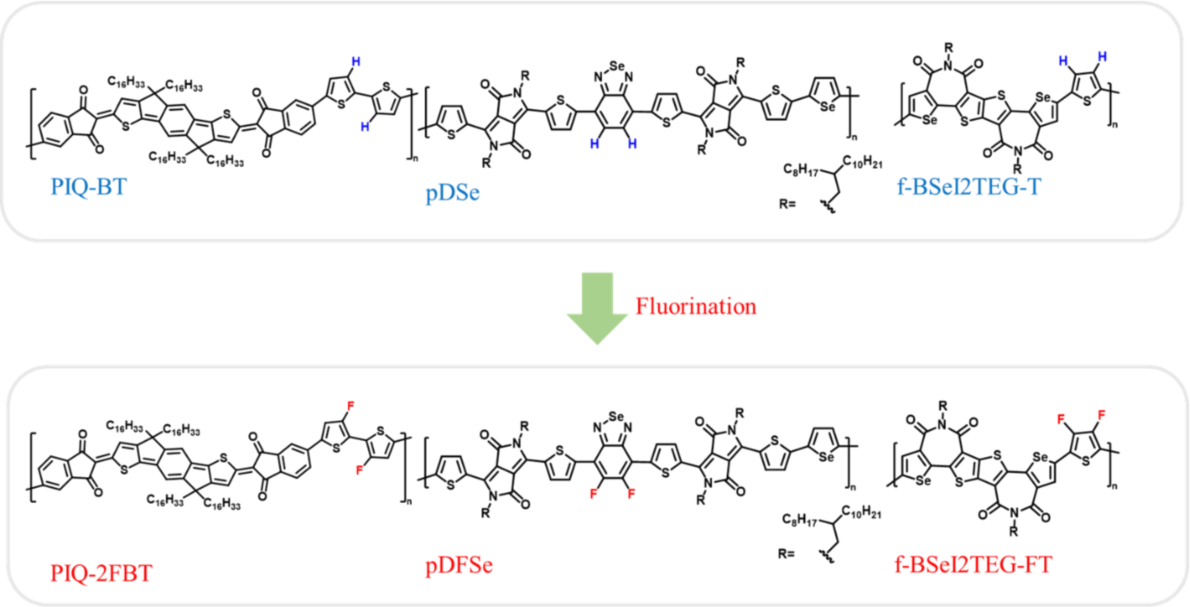

Figure S21. The chemical structure of n-type CPs with (without) fluorine (F) from literature.

Table S3 Thermoelectric parameters of n-type polymers with and without fluorine.

**Materials**	**Dopant**	**σ** (S cm $^{-1}$)	**S** (μV K^{-1})	**PF** (μW $m^{-1}K^{-2}$)	**Reference**
PIQ-BT	N-DMBI	2.49×10^{-3}	-1261.7	0.4	Ref.8
**PIQ-2FBT**	N-DMBI	1.05×10^{-2}	**-958.2**	0.96	Ref.8
pDSe	N-DMBI	5.9	-217.9	27.8	Ref.9
**pDFSe**	N-DMBI	62.6	**-145.8**	133.1	Ref.9
f-BSeI2TEG-T	N-DMBI	11.0	-111.7	8.8	Ref.10
**f-BSeI2TEG-FT**	N-DMBI	103.5	**-89.3**	70.1	Ref.10

Note that the introduction of fluorine atoms always enhances the Seebeck coefficient, at least to some degree, for p-type thermoelectric materials (see Table S2). However, the situation differs for n-type materials due to the challenges posed by n-type doping. We attribute the effect of fluorination on the reported n-type materials to the downward shift of the LUMO level, which significantly enhances the doping efficiency and reduces the gap between the Fermi energy and the transport energy. This results in a marked increase in electrical conductivity, but also in a corresponding decrease in the Seebeck coefficient, see Table S3.

S9 –Summary of mobilities for CPs with 1D and 2D structure in literatures

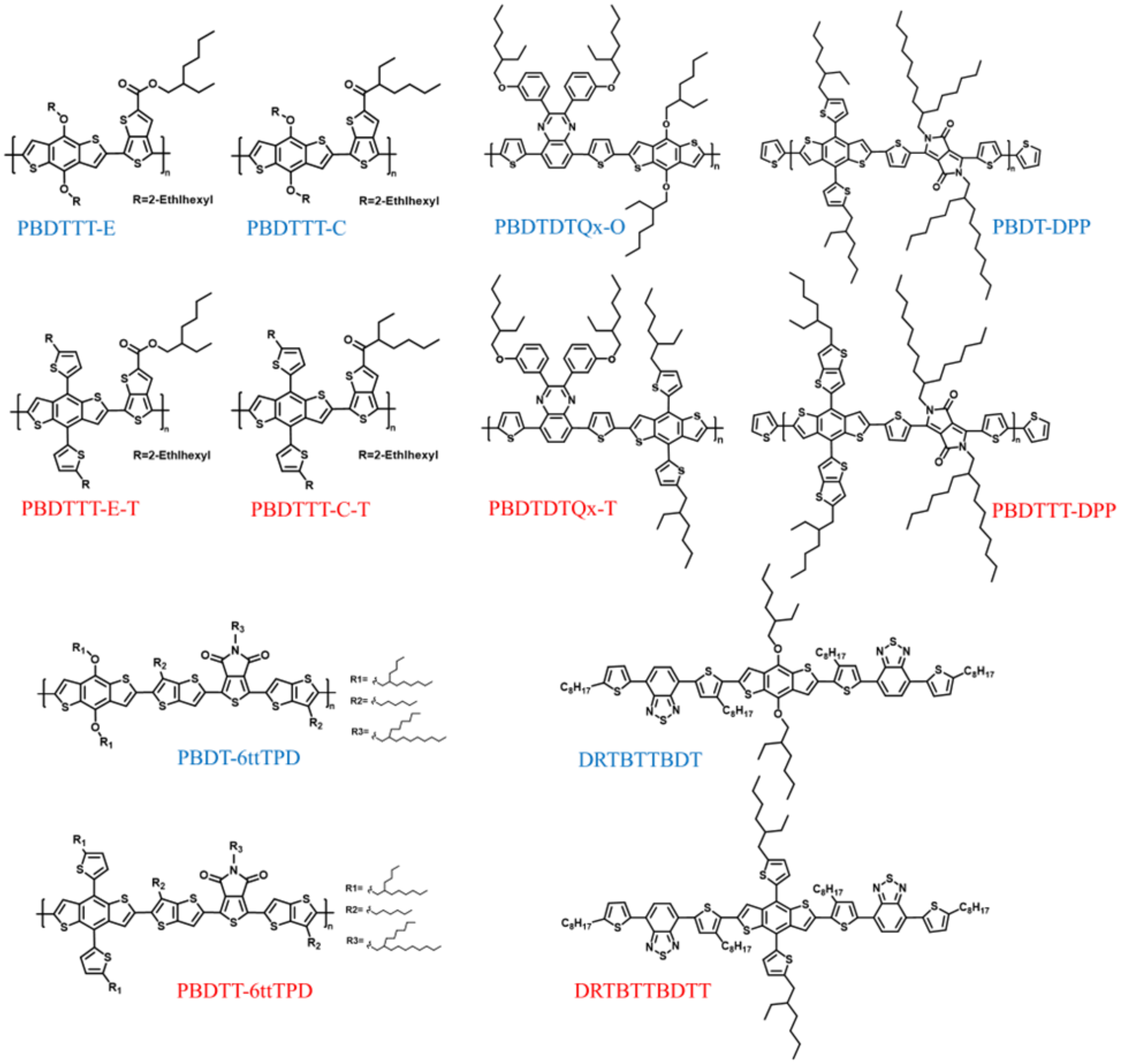

Figure S22. The chemical structure of CPs with 1D and 2D structure in literature. Corresponding mobilities are given in Table S4.

Table S4. Mobilities for CPs with backbone extended from 1D to 2D structure. The corresponding structures are given in Figure S22.

**Materials**	**Mobility** ($cm^2 V^{-1}s^{-1}$)	**Reference**
PBDTTT-E[a]	1.5×10^{-3}	Ref.11
**PBDTTT-E-T**[a]	**6.74×10^{-3}**	Ref.11
PBDTTT-C[a]	5.53×10^{-4}	Ref.11
**PBDTTT-C-T**[a]	**2.7×10^{-1}**	Ref.11
PBDTDTQx-O[a]	4×10^{-5}	Ref.12
**PBDTDTQx-T**[a]	**1.04×10^{-4}**	Ref.12
PBDT-6ttTPD[a]	1.17×10^{-4}	Ref.13
**PBDTT-6ttTPD**[a]	**4.21×10^{-4}**	Ref.13
DRTBTTBDT[b]	4.3×10^{-4}	Ref.14
**DRTBTTBDTT**[b]	**1.2×10^{-3}**	Ref.14
PBDT-DPP[a]	1.1×10^{-4}	Ref.15
**PBDTTT-DPP**[a]	**2.4×10^{-4}**	Ref.15

[a] Measured by using the space-charge-limited current (SCLC) method. [b] Measured by using the organic field-effect transistor (OFET) method.

References

[1] N. Felekidis, A. Melianas, M. Kemerink, *Org. Electron.* 2018, *61*, 318.
[2] S. Wu, W. Xing, M. Zhu, Y. Zou, Y. Sun, W. Xu, D. Zhu, *J. Mater. Chem. C* 2021, *9*, 4158.
[3] J.-F. Ding, G.-L. Chen, P.-H. Liu, K.-W. Tseng, W.-N. Wu, J.-M. Lin, S.-H. Tung, L. Wang, C.-L. Liu, *J. Mater. Chem. A* 2024, *12*, 9806.
[4] X. Geng, T. Du, C. Xu, Y. Liu, Y. Deng, Y. Geng, *Adv. Funct. Mater.* 2023, *33*, 2300809.
[5] E. H. Suh, Y. J. Jeong, J. G. Oh, K. Lee, J. Jung, Y. S. Kang, J. Jang, *Nano Energy* **2019**, *58*, 585.
[6] H.-P. Li, C.-Y. Gao, Z.-P. Chen, X.-H. Fan, L.-M. Yang, *Synth. Met.* **2024**, *301*, 117518.
[7] C.-H. Tsai, Y.-C. Lin, W.-N. Wu, S.-H. Tung, W.-C. Chen, C.-L. Liu, *J. Mater. Chem. C* **2023**, *11*, 6874.
[8] T. Du, Y. Liu, Y. Deng, Y. Geng, *Chin. J. Chem.* **2023**, *41*, 776.
[9] T. Shen, D. Liu, J. Zhang, Z. Wei, Y. Wang, *Angew. Chem., Int. Ed.* **2024,** *n/a*, e202409018.
[10] J. Li, M. Liu, K. Yang, Y. Wang, J. Wang, Z. Chen, K. Feng, D. Wang, J. Zhang, Y. Li, H. Guo, Z. Wei, X. Guo, *Adv. Funct. Mater.* **2023**, *33*, 2213911.
[11] L. Huo, S. Zhang, X. Guo, F. Xu, Y. Li, J. Hou, *Angew. Chem.* **2011**, *123*, 9871.
[12] R. Duan, L. Ye, X. Guo, Y. Huang, P. Wang, S. Zhang, J. Zhang, L. Huo, J. Hou, *Macromolecules* **2012**, *45*, 3032.
[13] J.-H. Kim, S. Wood, J. B. Park, J. Wade, M. Song, S. C. Yoon, I. H. Jung, J.-S. Kim, D.-H. Hwang, *Adv. Funct. Mater.* **2016**, *26*, 1517.
[14] Y. Chen, Y. Yan, Z. Du, Q. Liu, V. A. L. Roy, M. Sun, R. Yang, C. S. Lee, *J. Mater. Chem. C* **2014**, *2*, 3921-3927
[15] Y. Li, C.-Y. Chang, Y. Chen, Y. Song, C.-Z. Li, H.-L. Yip, A. K.-Y. Jen, C. Li, *J. Mater. Chem. C* **2013**, *1*, 7526.
